# Core outcome set for studies evaluating interventions to prevent or treat delirium in long-term care older residents: international key stakeholder informed consensus study

**DOI:** 10.1093/ageing/afae227

**Published:** 2024-10-13

**Authors:** Gregor Russell, Namrata Rana, Siobhan T Reilly, Anas Shehadeh, Valerie Page, Najma Siddiqi, Louise Rose

**Affiliations:** Osprey House, Lynfield Mount Hospital, Bradford District Care NHS Foundation Trust, Heights Lane, Bradford BD9 6PD, United Kingdom; City Hospital campus, Nottingham University Hospitals NHS Trust, Hucknall Rd, Nottingham NG5 1PB, United Kingdom; Centre for Applied Dementia Studies, Faculty of Health Studies, University of Bradford, Richmond Rd, Bradford BD7 1DP, United Kingdom; Centre for Applied Dementia Studies, Faculty of Health Studies, University of Bradford, Richmond Rd, Bradford BD7 1DP, United Kingdom; Department of Critical Care, Watford General Hospital, Vicarage Rd, Watford, Hertfordshire WD18 0HB, United Kingdom; Department of Health Sciences, University of York—Medical School Siwards Way, Heslington, York YO10 5DD, United Kingdom; Faculty of Nursing, Midwifery and Palliative Care, King's College London, 57 Waterloo Rd, London SE1 8WA, United Kingdom

**Keywords:** delirium, outcomes, interventions, core outcome set, long-term care, older people

## Abstract

**Background:**

Trials of interventions to prevent or treat delirium in older adults resident in long-term care settings (LTC) report heterogenous outcomes, hampering the identification of effective management strategies for this important condition. Our objective was to develop international consensus among key stakeholders for a core outcome set (COS) for future trials of interventions to prevent and/or treat delirium in this population.

**Methods:**

We used a rigorous COS development process including qualitative interviews with family members and staff with experience of delirium in LTC; a modified two-round Delphi survey; and virtual consensus meetings using nominal group technique. The study was registered with the Core Outcome Measures in Effectiveness Trials (COMET) initiative (https://www.comet-initiative.org/studies/details/796).

**Results:**

Item generation identified 22 delirium-specific outcomes and 32 other outcomes from 18 qualitative interviews. When combined with outcomes identified in our earlier systematic review, and following an item reduction step, this gave 43 outcomes that advanced to the formal consensus processes. These involved 169 participants from 12 countries, and included healthcare professionals (121, 72%), researchers (24, 14%), and family members/people with experience of delirium (24, 14%). Six outcomes were identified as essential to include in all trials of interventions for delirium in LTC, and were therefore included in the COS. These are: ‘delirium occurrence’; ‘delirium related distress’; ‘delirium severity’; ‘cognition including memory’, ‘admission to hospital’ and ‘mortality’.

**Conclusions:**

This COS, endorsed by the American Delirium Society and the European and Australasian Delirium Associations, is recommended for use in future clinical trials evaluating delirium prevention or treatment interventions for older adults residing in LTC.

## Key Points

- Delirium is a common occurrence in people resident in long-term care.
- There is little evidence available on interventions that can prevent or treat delirium in this setting.
- Following the Core Outcome Measures in Effectiveness Trials guidelines, we have developed a Core Outcome Set for use in delirium interventional trials in long-term care.
- We recommend this is used to improve homogeneity of outcome selection, supporting identification of effective interventions.

## Introduction

Delirium is characterized by fluctuating mental status with marked inattention and other cognitive disturbances [1]. In older adults, delirium is associated with serious negative outcomes including neurocognitive disturbance and cognitive decline [2], decreased functional status [3], and adverse events such as falls [4], and death [5]. There is moderate certainty evidence supporting the use of multicomponent interventions to prevent delirium in acute hospital settings, with a reduction in incidence of delirium by 43% reported [6], but no evidence for effectiveness of pharmacological interventions to treat delirium in non- acute hospital settings [7, 8]. Despite widespread prevalence, and the substantial impact on healthcare systems, there is limited evidence for effective prevention, and none for effective treatment [9], of delirium in older adults in other settings. A review of delirium prevention specifically in long-term care identified only three trials that met criteria for inclusion [10], and only one of these provided evidence of reduced incidence of delirium in those who received the intervention [11].

Lack of consensus on which outcomes should be selected when undertaking trials to evaluate an intervention makes evidence synthesis to arrive at clinical recommendations challenging [12]. A 2019 delirium think tank established the need for core outcome sets (COS) for studies of interventions to prevent and/or treat delirium as a research priority [13]. Core outcomes sets are an agreed-upon minimum set of outcomes to be measured and reported in all studies relating to a specific health condition [14]. If implemented, they should lead to reduced heterogeneity of trial outcome selection, and therefore support effective data synthesis. The Del-COrS project set out to establish four COS for trials of prevention and/or treatment of delirium using rigorous international consensus processes [15]. To-date, our group has established COS for delirium experienced by patients in critical care [16], in acute hospital care [17] and in palliative care [18]. The final COS relates to research on interventions to prevent and/or treat delirium experienced by older adults resident in long-term care settings (LTC), described herein.

Long-term care is considered to be an individual’s usual place of residence, in contrast to more temporary arrangements such as respite care, intermediate care and post-acute care [10]. While there is international variation in the way terminology is used [19], LTC is generally used to refer to institutional settings such as residential care or nursing homes. In the former, support with personal cares, help with daily living activities and supervision of medications is provided to residents by staff who generally do not hold a professional registration. In the latter least one staff member who is a registered healthcare professional (usually nursing) must be present at all times [20]. The characteristics of people resident in each type of these institutions will differ. Those in nursing homes will generally have greater complexity and higher levels of healthcare need. LTC residents commonly have factors which predispose to delirium, including long term physical conditions and dementia [21]. This leads to delirium being an important healthcare-related event in this setting, with prevalence rates of up to 37% reported [22]. Long-term care residents frequently experience negative outcomes if delirium develops, with six-month mortality rates of 37% described [23].

## Methods

To develop a COS for trials of interventions to prevent and/or treat delirium for older adults resident in LTC we followed the Core Outcome Measures in Effectiveness Trials (COMET) guidelines [24]. We report development of this COS in accordance with Core Outcome Set–STAndards for Reporting guidelines [25]. We commenced item generation to identify outcomes for inclusion in our consensus building processes through a systematic review we published previously [26]. We then conducted semi-structured interviews with key stakeholders followed by a two-step consensus building process, comprising a two-round international, web-based Delphi and two virtual consensus workshops.

### Participant recruitment

Using purposive sampling, we sought an international sample from three stakeholder groups who had experience of delirium in LTC settings: [1] researchers; [2] healthcare professionals and paid caregivers; and [3] people who had lived experience of delirium in a LTC setting, including family members of LTC residents. We only recruited those able to read English as we did not have sufficient resources to translate the Delphi materials into other languages. For qualitative interviews, we recruited participants representing stakeholder groups 2 and 3 using a multi-modal strategy involving personal and professional contacts, snowballing, and support from NHS Local Clinical Research Networks in the form of direct approaches to potential participants. We continued to recruit until we considered sufficient information power [27] was achieved i.e. no new outcomes were being suggested.

For the Delphi panel, we used a similar multi-modal recruitment strategy also recruiting from the NHS ‘Join Dementia Research’ register, through promotion by the American Delirium Society, Australasian Delirium Association, European Delirium Association, British Geriatric Society, and European Geriatric Medicine Society, and via NHS Futures platform, Twitter (now X) and Carers’ support groups. The lead investigator also contacted all corresponding authors of studies identified via our systematic review [26]. All participants completing the Delphi survey were invited to take part in the consensus workshop meetings, held virtually using Microsoft Teams.

### Semi-structured interviews

Two interviewers conducted semi-structured telephone interviews focusing on the participant’s experience of delirium and outcomes they considered important to measure. The interview guides used are shown in the supplementary materials, Appendix 1. Interviews were digitally recorded and professionally transcribed. We used directed content analysis [28] to identify outcomes for potential inclusion in the COS. Interview findings were analysed independently by two researchers with final decisions discussed with a third researcher.

### Modified Delphi methods

We reduced items generated from the systematic review and interview analyses removing redundant, aggregate population, and feasibility or process outcomes. We then reviewed outcome wording for clarity and grouped them into domains using the COMET taxonomy [29]. We used the bespoke DelphiManager software, Version 4 (COMET Initiative, Liverpool, UK) to administer Delphi rounds. Participants were asked to self-select their preferred key stakeholder group (i.e. person with lived experience of delirium; healthcare professional; researcher) and to rate the importance of each outcome for COS inclusion without consideration of measurability or feasibility. We used the 9-point Grading of Recommendations Assessment, Development and Evaluations (GRADE) Scale [24] for importance ratings. Scores of 1 to 3 were considered not important; 4 to 6 important but not critical; and 7 to 9 as critical for inclusion. This scoring system, recommended by COMET, facilitates maximum discrimination between questionnaire items [24]. Participants were provided an ‘Unable to Score’ response option and the opportunity to suggest additional outcomes perceived as missing from the outcomes provided. To avoid presentation bias, outcome domain presentation order was randomized using the DelphiManager software.

Upon completion of each Delphi Round, we determined the proportion of participants rating each outcome with scores of 7 to 9, 4 to 6, and 1 to 3 for the entire Delphi panel, and separately, for each of our three stakeholder groups. The study team reviewed additional outcomes suggested for inclusion in Round 2. Participants who completed Round 1 were invited to participate in Round 2. Participants received their own Round 1 scores and the summarized scores, with visual representation for each stakeholder group using histograms of each outcome. Participants were asked to re-score outcome importance based on this feedback. For both Rounds, we sent three completion reminders by email via the DelphiManager software.

### Consensus workshops

Due to the challenges of holding face-to-face meetings following the Covid-19 pandemic, and to ensure accessibility to all stakeholder groups, we held two consensus workshops using Microsoft Teams, facilitated by authors and supported by their colleagues (see acknowledgements). Outcomes brought to the consensus workshops met the following criteria, as recommended by COMET [23]: rated across all respondents as ‘critical for inclusion’ by >70%, and ‘not important’ by <5%. Using nominal group technique methods [30], supported by Google Jamboard (a digital whiteboard for real-time collaboration), we held iterative rounds of small and whole group discussion. During these workshops we identified outcomes where consensus was reached to include or exclude from the COS. For outcomes where consensus was not achieved, we contacted workshop participants and asked them to vote via email on whether the outcome should be included. An outcome was included in the COS if more than 50% of respondents voted in favour.

The study protocol and systematic review results have been published previously [15, 26], and the project was registered with the COMET initiative (https://www.comet-initiative.org/studies/details/796).

## Results

During item generation, our systematic review (18 studies recruiting 5639 participants) identified 12 delirium-specific outcomes and 25 non delirium-specific outcomes within 13 COMET taxonomy categories [26]. We recruited 13 healthcare professionals and five family members (N = 18) to participate in qualitative interviews. We identified 54 potential outcomes, of which 44 were not identified in our systematic review. These are shown in Table 1. Item reduction resulted in 43 outcomes for the Delphi Round 1. The process of outcome reduction is shown in Table 2.

**Table 1:** Outcomes identified by interview participants

Outcome (N = 18 participants)	n (%)
Social interactions (with other residents, care staff and family members)	13 (72)
Food intake	12 (67)
Staff knowledge of delirium (recognition/management of delirium)	12 (67)
Cognitive functioning (memory, attention, perception, recognition, disorientation)	12 (67)
Engagement with meaningful activities (e.g. hobbies)	12 (67)
Fluid intake	11 (61)
Falls (number of falls/risk of falls/severity of falls)	11 (61)
Agitation	10 (56)
Performance of activities of daily living	10 (56)
Family awareness of delirium	8 (44)
Psychotic symptoms (hallucinations, delusions, paranoia)	8 (44)
Sleep	7 (39)
Aggression (verbal and physical)	6 (33)
Happiness/life satisfaction	5 (28)
Duration of delirium	5 (28)
Confusion	5 (28)
Distress	5 (28)
Patient awareness of delirium	4 (22)
Public awareness of delirium	4 (22)
Use of medications (e.g. antipsychotics and benzodiazepines)	4 (22)
Number of episodes of delirium	3 (17)
Admission to hospital	3 (17)
Mobility	3 (17)
Dementia (subsequent diagnosis/worsening of existing dementia)	3 (17)
Pain	3 (17)
Infections	3 (17)
Self-harm	2 (11)
Harm to others	2 (11)
Mortality rate	2 (11)
Mood	2 (11)
Quality of life	2 (11)
Family’s perception of quality of care	2 (11)
Delirium severity	2 (11)
Delirium resolution	2 (11)
Anxiety	2 (11)
Presence of a treatment plan	2 (11)

In addition, the following outcomes were identified by only one interview participant: awareness of delirium in primary care; awareness of delirium in care homes; frequency of delirium episodes; long lasting effects of delirium; stress levels; family carers’ opinions of recovery from delirium; evidence of treatment plan being followed; frequency of contact with secondary care; weight loss; frequency of reportable events; skin integrity; constipation; continence; return to normal routine; communication skills; improvement in relationship between LTC resident and health care professionals; engagement with/acceptance of care provider; and improvement in inflammatory markers

**Table 2:** Outcome reduction process

Outcome	Source	Item Reduction	Reason for Redundancy	Final Delphi Wording
Delirium incidence	SR	INC-MOD		Delirium occurrence
Delirium prevalence	SR	REDUN	Due to variable use of terms prevalence & incidence combined as delirium occurrence	
Delirium severity	SR + IV	INC		Delirium severity
Cognitive functioning	SR + IV	INC-MOD		Cognition including memory
Delirium duration	SR + IV	INC		Delirium duration
Nurse knowledge of delirium	SR	REDUN	Considered to overlap with staff awareness/understanding of delirium	
Agitation	SR + IV	INC-MOD		Agitation occurrence
Delirium motoric subtype	SR	INC-MOD		Delirium type
Use of antipsychotic medication	SR	INC-MOD		Use of antipsychotic or sedative medication
Number of delirium episodes	SR + IV	INC		Number of delirium episodes
Nurse confidence in managing delirium	SR	REDUN	Considered to overlap with staff awareness/understanding of delirium	
Nurse ability to recognize delirium	SR	REDUN	Considered to overlap with staff awareness/understanding of delirium	
Admission to hospital	SR + IV	INC		Admission to hospital
Mobility and falls	SR	REDUN	Separated into two existing outcomes	
Performance of ADLs	SR + IV	INC-MOD		Ability to perform activities of daily living
Mortality	SR + IV	INC		Mortality
Quality of life	SR + IV	INC-MOD		Health-related quality of life
Infections	SR + IV	INC		Infection
Health and social care resource use	SR	INC		Health and social care resource use
Hydration	SR	REDUN	Considered to overlap with fluid intake	
Polypharmacy	SR	INC		Polypharmacy
Medication appropriateness	SR	INC		Medication appropriateness
Number of contacts with Primary care	SR	REDUN	Considered to overlap with Health and social care resource use	
Malnutrition	SR	REDUN	Considered to overlap with food intake	
Depressive symptoms	SR	INC		Depressive symptoms
Quality of interprofessional communication	SR	INC		Quality of interprofessional communication
Acceptability of and satisfaction with intervention	SR	REDUN	Considered to overlap with Family’s perception of quality of care	
Staff knowledge of delirium	IV	INC-MOD		Staff awareness and/or understanding of delirium
Family awareness of delirium	IV	INC-MOD		Public awareness and/or understanding of delirium
Psychotic symptoms	IV	INC		Psychotic symptoms
Aggression (verbal and physical)	IV	INC-MOD		Aggression
Confusion	IV	REDUN	Considered to overlap with cognitive functioning	
Distress	IV	INC-MOD	Separated into three different outcomes	Patient Distress AND Family/carer distress AND Staff distress
Long term care resident awareness of delirium	IV	REDUN	Considered to overlap with Public awareness and/or understanding of delirium	
General public awareness of delirium	IV	REDUN	Considered to overlap with Public awareness and/or understanding of delirium	
Use of medication	IV	REDUN	Considered to overlap with Use of antipsychotic or sedative medication	
Delirium resolution	IV	INC		Delirium resolution
Anxiety	IV	REDUN	Considered to overlap with Patient Distress	

(*Continued*)

**Table 2:** Continued

Outcome	Source	Item Reduction	Reason for Redundancy	Final Delphi Wording
Awareness of delirium in primary care	IV	REDUN	Considered to overlap with Staff awareness and/or understanding of delirium	
Awareness of delirium in care homes	IV	REDUN	Considered to overlap with Staff awareness and/or understanding of delirium	
Frequency of delirium	IV	REDUN	Considered to overlap with Number of delirium episodes	
Long lasting effects of delirium	IV	REDUN	Considered to overlap with Development or worsening of dementia	
Level of stress	IV	REDUN	Considered to overlap with Patient distress	
Family carer’s opinions on resident’s recovery from delirium	IV	REDUN	Considered to overlap with delirium resolution	
Social interactions	IV	INC		Social interactions
Food intake	IV	INC		Food intake
Engagement with meaningful activities	IV	INC		Engagement with meaningful activities
Fluid intake	IV	INC		Fluid intake
Falls (number of falls)	IV	INC		Falls
Sleep	IV	INC		Sleep
Happiness/life satisfaction	IV	INC		Happiness/life satisfaction
Mobility	IV	INC		Mobility
Development/worsening of dementia	IV	INC		Development/worsening of dementia
Pain	IV	INC		Pain
Self-harm	IV	INC		Self-harm
Harm to others	IV	REDUN	Considered to overlap with aggression	
Mood	IV	REDUN	Considered to overlap with happiness/life satisfaction	
Family’s perceptions of quality of care	IV	INC		Family’s perceptions of quality of care
Presence of treatment plan	IV	PROC		
Improvement in inflammatory markers	IV	PROC		
Number of contacts with secondary care	IV	REDUN	Considered to overlap with Health and social care resource use	
Weight loss	IV	INC		Weight loss
Number of reportable incidents	IV	REDUN	Considered to overlap with Aggression and Falls	
Skin integrity	IV	INC		Skin integrity
Constipation	IV	INC		Constipation
Continence	IV	INC		Continence
Return to normal routine	IV	REDUN	Considered to overlap with delirium resolution	
Communication skills	IV	REDUN	Considered to overlap with Staff awareness and/or understanding of delirium	
Improvement in relationship between resident and care staff	IV	REDUN	Considered to overlap with Care and treatment interference	
Engagement/acceptance of care and treatment provided	IV	INC-MOD		Care and treatment interference
Evidence treatment plan followed	IV	PROC		

SR = identified in systematic review

IV = identified in interviews

INC = included in Delphi

INC-MOD = included in Delphi but with modified wording

REDUN = redundant, considered to conceptually overlap with another outcome

PROC = process outcome

We recruited 169 participants from 12 countries for Delphi round one. 121(72%) identified primarily as healthcare professionals, 24 (14%) primarily as LTC residents who had experienced delirium/family members, and 24 (14%) primarily as researchers. Delphi participant characteristics are shown in Table 3. In round one, of the 43 outcomes considered, 26 (60%) met criteria for COS inclusion when considering all participant responses (see Supplementary Material Appendix 2). Of the 169 participants, 107 (63%) took part in round two. These participants comprised 78 (73%) clinicians, 14 (13%) LTC residents who had experienced delirium/family members and 15 (14%) researchers. Of the 43 outcomes rated by participants in Round 2, 25 (58%) met for inclusion in the COS considering all participant responses, with 12 (28%) rated as meeting criteria by all three stakeholder groups (Table 4).

**Table 3:** Delphi participant characteristics

	n (%)
Country of residence N = 169	
United Kingdom	146 (86.4)
Europe other than UK	9 (5.3)
USA	6 (3.5)
Australia	2 (1.2)
Other	6 (3.5)
Delirium role N = 169	
Clinical only	71 (42)
Research and clinical	53 (31.4
Delirium survivor/family member	22 (13)
Family member with research and clinical roles	12 (7.1)
Research only	11 (6.5)
Profession (clinicians) N = 136	
Physician	58 (42.6)
Nurse/nurse practitioner	50 (36.8)
Health care assistant	15 (11)
Allied health professional	6 (4.4)
Other	7 (5.1)
Years of experience (clinicians) N = 136	
More than 10 years	116 (85.3)
6–10 years	8 (5.9)
Less than 5 years	12 (8.8)

Clinicians include those who identified primarily as clinicians, but also 15 participants whose primary identification was as a researcher, but who also reported having a clinical role

**Table 4:** Results from Delphi round two

Outcomes (% rate as critical for inclusion)	Overall	Researcher (N = 15)	Clinician (N = 78)	Family (N = 14)
Delirium severity	95.1	93.3	96.0	92.3
Delirium resolution	95.1	93.3	94.5	100.0
Patient distress	92.2	100.0	89.3	100.0
Agitation occurrence	90.4	93.3	90.8	84.6
Delirium duration	90.1	93.3	90.4	84.6
Delirium occurrence	89.1	93.3	86.5	100.0
Aggression	88.5	73.3	92.1	84.6
Development/worsening dementia	88.5	80.0	89.5	92.3
Falls	88.4	80.0	92.0	76.9
Number of delirium episodes	88.2	86.7	89.2	84.6
Psychotic symptoms	88.0	73.3	89.8	100.0
Sleep	87.5	80.0	88.7	92.3
Staff awareness of delirium	87.4	73.3	88.0	100.0
Mortality	86.1	93.3	85.3	81.8
Cognition including memory	85.6	86.6	86.8	76.9
Medication appropriateness	85.0	78.7	85.1	91.7
Fluid intake	84.3	86.6	86.7	66.7
Infection	83.3	66.7	85.1	92.3
Pain	83.3	80.0	86.7	66.7
Admission to Hospital	82.4	86.7	81.3	81.3
Quality of interpersonal communication	81.2	66.7	82.2	92.3
Use of antipsychotic medication	80.4	93.3	75.7	92.3
Polypharmacy	74.5	71.4	78.1	54.6
Health related quality of life	73.8	53.3	79.0	75.0
Lack of cooperation with care	72.8	40.0	76.0	92.3
Self-harm^a^	72.0	66.7	71.2	83.3
Ability to perform ADLs	68.3	66.7	69.7	61.5
Depressive symptoms	68.3	40.0	71.1	84.6
Family/carer distress	65.7	60.0	65.3	75.0
Happiness/life satisfaction	63.5	40.0	64.5	84.6
Mobility	63.5	60.0	64.5	61.5
Change in continence	61.5	26.7	66.2	75.0
Food intake	60.6	53.3	61.8	61.5
Delirium subtype	58.8	40.0	63.5	53.6
Health/social care resource use	56.3	26.7	61.3	61.5
Public awareness of delirium	53.9	26.7	56.0	75.0
Family perception of quality of care	53.4	33.3	50.7	92.3
Weight loss	51.0	20.0	56.0	58.3
Constipation	47.6	13.3	52.0	61.5
Engagement with meaningful activities	44.2	40.0	43.4	53.6
Social interactions	44.2	26.7	43.4	69.2
Staff distress	44.1	26.7	43.2	69.2
Altered skin integrity	29.7	20.0	31.1	33.3

a Self-harm did not meet the inclusion criterion that less than 5% of respondents should rate it as ‘not important’.

In the 26 outcomes rated as ‘critical to include’ by >70% of respondents in round one, the proportion of respondents rating as them ‘critical to include’ in round two increased, except for the outcome ‘public awareness of delirium’. The proportion rating this outcome as ‘critical for inclusion’ dropped from 83% to 54%. For outcomes rated by <70% of respondents as ‘critical for inclusion’ in round 1, nine had a reduction in the proportion of participants providing this rating and eight outcomes had an increase. Only one outcome, ‘lack of co-operation with care’, received an increase in support which took it over the 70% inclusion threshold. One outcome, ‘self-harm’, failed to meet the inclusion criterion on the proportion of candidates rating it as ‘not important’ to include in the COS.

**Figure 1:**
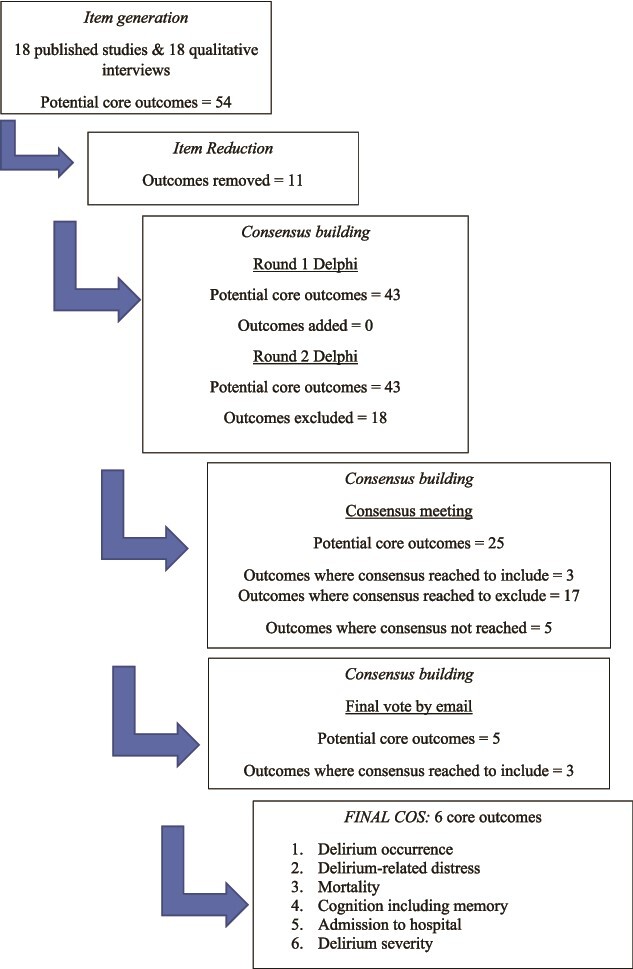
Development of COS for trials of interventions to prevent or treat delirium in older adults in long-term care

Nineteen experts (including four LTC residents/family members) participated in the two consensus meetings. For inclusion in the COS, the members of both groups had to independently decide that the outcome should be included. Agreement was reached during meetings on 20 outcomes. There was consensus to include three outcomes in the COS: ‘delirium occurrence’, ‘mortality’ and ‘delirium-related distress’. In 17 cases, there was agreement to exclude the outcome. This included six outcomes which were felt by one or both groups to be subordinate to another outcome. There was disagreement between the two group over five outcomes: in these cases, one group felt the outcome should be included, and the other felt it should be excluded, or was subordinate. These outcomes were put to all consensus meeting participants in a round of email voting on whether to include in the COS. Three further outcomes were included following this email vote: ‘delirium severity’, ‘cognition including memory’ and ‘admission to hospital’. The decision making that underpinned this process is shown in Supplementary Materials Appendix 3, and the final LTC delirium COS is shown in Figure 1.

## Discussion

This study employed rigorous methodology recruiting a panel of international experts to develop a COS comprising six outcomes to be used in all future trials of interventions to prevent and/or treat delirium in older adults resident in LTC. Outcomes that achieved consensus were ‘delirium occurrence’; ‘delirium-related distress’; ‘delirium severity’; ‘cognition including memory’; ‘admission to hospital’ and ‘mortality’. This COS completes the Del-COrS programme of work, with COS now available for delirium interventional research in LTC, critical care [16], acute hospital care [17], and palliative care [18] settings. There are similarities across these COS, with the outcomes ‘delirium occurrence’, and ‘emotional distress’ or ‘delirium-related distress’, common to all.

‘Delirium occurrence’ is a composite measure including both delirium incidence and prevalence. Measurement of delirium occurrence is fundamental to determining the effectiveness of interventions designed to prevent delirium. This outcome was introduced at the COS item reduction phase of all four COS due to the challenges of accurately determining the timing of onset of delirium, and thus delirium incidence. This is particularly pertinent to the older adult LTC population who have high rates of underlying dementia [23] making delirium detection challenging.

The outcome ‘delirium-related distress’ was considered critically important for inclusion at the conclusion of the Delphi rounds but was the focus of much debate during the consensus meetings. Debate focused on whether several of the outcomes were conceptually independent of the outcome ‘delirium-related distress’. Consensus was reached was that ‘agitation’ ‘aggression’, ‘use of antipsychotic and sedative medication’ and ‘lack of cooperation with care and treatment’ were subordinate to ‘delirium related distress’, while ‘delirium severity’ and ‘psychotic symptoms’ preserved independent status.

There are important differences in the four COS reflecting differing stakeholder priorities relating to population characteristics. For example, the outcome ‘mortality’ voted for inclusion in this LTC COS is only shared with the critical care COS. Mortality is also included in a number of other critical care related COS [31, 32]. Inclusion of mortality in the LTC COS reflects the clinical significance of delirium occurring in this population, in whom mortality rates of 25% within 1 month of developing delirium have been documented [33]. The outcome ‘admission to hospital’ is unique to the LTC COS. This is unsurprising when comparing to acute hospital and critical care COS which reflect in-patient populations. It could be argued that admission to hospital may be a positive event for some people who develop delirium in LTC, allowing access to more intensive treatment for the underlying cause of the delirium. Set against this, in the LTC population, transitions in care are frequently negative events and associated with preventable decline in health status and adverse care incidents such as medication errors [34]. On balance, interventions which reduce the occurrence of admissions to hospital would be desirable, as long as they did not lead to worse performance on other core outcomes such as mortality, delirium severity and delirium-related distress.

Two outcomes ‘delirium duration’ and ‘time to delirium resolution’, included in both the acute and critical care COS, were not included in this LTC COS. This may reflect the challenges of determining when delirium has resolved in a population where dementia is very common, and where there is considerable overlap in symptoms between these conditions [34]. This issue was also debated by consensus workshop participants in terms of the outcome ‘cognition including memory’. Despite its final inclusion in the COS, workshop participants noted the challenge of reliably distinguishing between delirium and dementia as the cause of changes in cognition, which would impact on the ability to detect the effect of a delirium prevention or treatment intervention using this outcome. They nevertheless felt this to be sufficiently important to warrant inclusion despite these challenges around measurement.

The outcome ‘health-related quality of life’ is included in all three of the other delirium COS but found little support in the consensus workshop discussions in this study. This may reflect a view that those living in LTC, most of whom will have moderate to severe dementia [23], already have significantly impaired quality of life, although this is not borne out by evidence [35] suggesting little relationship between quality of life and dementia severity.

Strengths of this study include participation by family members with experience of delirium across all COS generation phases, a relatively large stakeholder panel with international representation, well-attended and representative consensus workshops, and adherence to COMET COS development methods. Study limitations include exclusion of non-English speaking research participants; and while the geographic variation in Delphi participants was reasonably broad, with representation from 12 countries, UK-resident participants dominated, accounting for over 80% of those responding. Moreover, clinicians were the largest category of Delphi respondents, and may have exerted disproportionate influence on outcome ratings. We did not ask Delphi participants to identify if they were a staff member of an LTC institution, as opposed to a healthcare professional from another organization providing services in LTC, and therefore cannot comment on potential differences in outcome priorities among these groups. Finally, we did not ask Delphi participants to self-identify as either a LTC resident who had experienced delirium or a family member, so we therefore cannot be sure the voice of LTC residents is adequately represented, with only one person who experienced delirium participating in a consensus meeting.

## Conclusion

With development of this COS, we seek to promote standardized outcome selection and reporting in future studies of interventions to prevent and/or treat delirium in older adults resident in LTC. We recommend that delirium researchers adopt this COS as part of future research protocols. This will improve person- and family-centeredness of outcome selection and homogeneity of reported outcomes. It will also increase statistical power, precision of meta-analyses, and the ability to make evidence-based decisions to improve the management of delirium for older adults resident in LTC where it is a common and potentially devastating occurrence. We hope dissemination of this COS will help draw attention to the relative paucity of delirium interventional research conducted in this population compared to other populations at risk of delirium, acting as a stimulus for further research in this area. Further work is now needed to operationally define the six core outcomes and to develop consensus in selecting validated measurement instruments.

## Supplementary Material

aa-24-1021-File002_afae227

Supplementary_material_Appendix_1_afae227

Supplementary_Materials_Appendix_2_afae227

Supplementary_Materials_Appendix_3_afae227

## Data Transparency and Availability Statement:

The data relating to outcomes identified from the interviews, and voting in both rounds of the Delphi consensus survey, and the steps taken to carry out the item reduction process, are presented in the manuscript or in the supplementary materials.
