# Supplementary material for: Core outcome set for studies evaluating interventions to prevent or treat delirium in long-term care older residents: international key stakeholder informed consensus study
Source: Age Ageing. 2024 Oct 13;53(10):afae227. doi: 10.1093/ageing/afae227 (PMC11471312; doi:10.1093/ageing/afae227)
Supplement: aa-24-1021-File002_afae227 [file aa-24-1021-file002_afae227.docx]

Core outcome set for studies evaluating interventions to prevent or treat delirium in long-term care older residents: international key stakeholder informed consensus study

Appendices

Appendix 1: Interview Topic Guides page 2

Appendix 2: Results from Delphi Round 1 page 8

Appendix 3: Outcomes of Consensus Meetings and Voting page 9

Supplementary Materials Appendix 1: Interview Topic Guides

DEVELOPMENT OF CORE OUTCOME SETS FOR EFFECTIVENESS TRIALS OF INTERVENTIONS TO PREVENT AND/OR TREAT DELIRIUM IN LONG TERM CARE (DEL-CORS)

INTERVIEW GUIDE: SURVIVORS AND FAMILY MEMBERS

INSTRUCTIONS

1. Introduce self and study to interviewee
2. Confirm consent still being given
3. Confirm maximum interview time of up to 1hr.
4. Indicate recording equipment and advise when switched to record.
5. Explain that research notes will be written too.

PREAMBLE

- We want to find out the important outcomes for survivors of delirium and their family members.
- In this interview, we want discuss outcomes that are important to people diagnosed with delirium and their care partners (family or unpaid carers).
- This research aims to identify which outcomes are most important from their perspectives as well as leaders working in this area.
- An ‘outcome’ is the result, effect, or impact, of support interventions or treatments.
- Outcomes can vary considerably.
- For example, outcomes include improvements in physical or mental health, satisfaction about provided services…
- For example, in a study of how well a new delirium treatment works, ‘outcomes’ might include: a measure of how fast the delirium goes away; how long a person had to stay in hospital because of delirium; whether a person hurt themselves because of delirium.
- Currently there are wide variations in reported outcomes making it difficult to combine and compare results.
- Therefore, this study aims to identify what should be measured and reported, as a minimum, when people experience delirium (or caring for a relative who is living with delirium).
- These ‘must-to-have’ (essential, important) outcomes can also be used in routine care, clinical audit, and research.

OPENING QUESTIONS

Can you please tell me about yourself?

…Demographics:

Patients/family members

Patients who survived delirium within the last 18 months and family members who had direct contact with patients while experiencing delirium within the last 18 months irrespective of survival, that is, we will interview family members of patients who did and did not survive the intensive care unit.

Age, years (≤65 and >65)

Sex (male and female)

Partner status (has partner and no partner)

Country of residence (North America, Europe/UK, Australasia and other)

Tell me about your experience with delirium.

…when was your relative diagnosed?

…what symptoms did your relative show?

Can you please tell me how delirium affected your life?

….your relative’s life?

What changed in your relative’s life because of the delirium?

What was the most important change? ...why?

How did you feel when your relative was experiencing delirium (was having an episode of delirium)?

…when it was gone? …why?

…what did you do?...why?

…what were you worried about (in the long term)?...why?

How would you describe your experience with delirium?

...when compared with other chronic illness you have?

…what is the difference?

What was the most important negative result/consequence/impact/effect/change delirium had on your relative?

What bothered your relative the most about having delirium?

What aspects of your life do you think were limited by the delirium?

Which was the most important aspect to you? …why?

When did you feel that you need help to deal with the symptoms of delirium?

What were the major improvements you experienced, in your life, when the delirium was gone?

What strategies did you use to cope with delirium?

…what was the most effective one? …why?

What advice you would give to someone in your situation?

MORE SPECIFIC QUESTIONS

Considering your experience, what outcomes would you want future studies to measure to ensure the most important aspects of this condition are considered when deciding how well an intervention works? …for example, when we have looked at published studies, we have found that many look at treatments to make the experience of delirium shorter.

…what other things can you think of that should be studied to determine whether treatments work?

…either to prevent delirium from happening altogether, or to treat delirium if it has occurred.

What do you think we should measure to determine the effectiveness of support interventions?

Is there anything else you’d like to talk about in relation research for treatments to prevent or treat delirium?

DEVELOPMENT OF CORE OUTCOME SETS FOR EFFECTIVENESS TRIALS OF INTERVENTIONS TO PREVENT AND/OR TREAT DELIRIUM IN LONG TERM CARE (DEL-CORS)

INTERVIEW GUIDE: CLINICAL STAFF

INSTRUCTIONS

1. Introduce self and study to interviewee.
2. Review consent to ensure still being given
3. Confirm maximum interview time of up to 1hr.
4. Indicate recording equipment and advise when switched to record.
5. Explain that research notes will be written too.

PREAMBLE

- We want to find out the important outcomes to measure in studies of interventions to prevent or treat delirium
- In this interview, we want discuss outcomes that are important to people who provide care for people who experience delirium in long term care
- An ‘outcome’ is the result, effect, or impact, of support interventions or treatments.
- Outcomes can vary considerably.
- For example, outcomes include improvements in physical or mental health, satisfaction about provided services…
- For example, in a study of how well a new delirium treatment works, ‘outcomes’ might include: a measure of how fast the delirium goes away; how long a person had to stay in hospital because of delirium; whether a person hurt themselves because of delirium.
- Currently there are wide variations in reported outcomes making it difficult to compare research findings and draw broader conclusions about effectiveness.
- Therefore, this study aims to identify what should be measured and reported, as a minimum, in all delirium clinical trials.
- These essential or core outcomes can also be used in routine care, clinical audit, and research other than randomised trials.

OPENING QUESTIONS

Can you please tell me about yourself?

…Demographics:

Expert clinicians

Physicians, nurses and allied health professionals who do not meet the criteria of a trialist.

Profession (physician, nurse and allied health professionals)

Years of relevant clinical experience (<5, 5–10 and >10)

Country of residence (North America, Europe/UK, Australasia and other)

Tell me about your experience with delirium.

…what are people usually concerned about when they are diagnosed with delirium?

…what about their family members?

Can you please tell me how delirium affect people who have it?

What changes happen to people because of the delirium?

What was the most important change? ...why?

…what about their family members?

What was the most important negative result/consequence/impact/effect/change delirium have on people?

…what do people who are living with delirium usually worry about (in the long term; in the short term)?...why?

What were the major improvements people experience when the delirium was gone?

How would you describe the symptoms of delirium?

...when compared with other chronic illness?

…what is the difference?

When do people usually ask for help to deal with the symptoms of delirium?

What strategies do you recommend using to cope with delirium?

…what was the most effective one? …why?

MORE SPECIFIC QUESTIONS

Considering your experience, what outcomes would you want future studies to measure to ensure the most important aspects of this condition are considered when deciding how well an intervention works?

…for example, when we have looked at published studies, we have found that many look at treatments to make the experience of delirium shorter.

…what other things can you think of that should be studied to determine whether treatments work?

…either to prevent delirium from happening altogether, or to treat delirium if it has occurred.

What do you think we should measure to determine the effectiveness of support interventions?

Is there anything else you’d like to talk about in relation research for treatments to prevent or treat delirium?

Supplementary Materials Appendix 2: Results from Delphi Round 1

| Outcomes (% rating critical to include) | Overall | Researcher  (N=24) | Clinician  (N=121) | Family  (N=24) |
| --- | --- | --- | --- | --- |
| Patient distress | 91.36 | 100.00 | 90.60 | 86.36 |
| Delirium severity | 88.68 | 86.36 | 89.57 | 86.36 |
| Agitation occurrence | 84.57 | 86.96 | 86.33 | 72.73 |
| Mortality | 84.41 | 81.82 | 83.33 | 94.44 |
| Delirium resolution | 84.28 | 81.82 | 86.96 | 72.73 |
| Delirium duration | 83.65 | 86.36 | 84.35 | 77.27 |
| Public awareness of delirium | 82.91 | 68.18 | 82.61 | 100.00 |
| Staff awareness of delirium | 82.91 | 68.18 | 82.61 | 100.00 |
| Aggression | 82.71 | 65.22 | 80.33 | 81.82 |
| Falls | 82.39 | 72.73 | 84.35 | 81.82 |
| Psychotic symptoms | 81.76 | 72.73 | 82.61 | 86.36 |
| Delirium occurrence | 81.65 | 86.36 | 81.58 | 77.27 |
| Cognition including memory | 81.24 | 81.81 | 84.35 | 60.87 |
| Development/worsening of dementia | 80.62 | 72.73 | 83.35 | 69.57 |
| Pain | 80.62 | 69.57 | 84.35 | 72.73 |
| Number of delirium episodes | 79.87 | 81.82 | 80.00 | 77.27 |
| Fluid intake | 76.73 | 65.22 | 79.13 | 76.19 |
| Use of antipsychotic medication | 75.95 | 81.81 | 77.39 | 61.90 |
| Medication appropriateness | 75.93 | 72.72 | 77.78 | 69.57 |
| Infection | 75.77 | 52.17 | 81.58 | 70.83 |
| Sleep | 74.54 | 72.72 | 77.59 | 60.87 |
| Quality of interpersonal communications | 73.01 | 63.63 | 73.73 | 78.26 |
| Polypharmacy | 71.34 | 61.90 | 79.31 | 35.00 |
| Admission to hospital | 70.89 | 73.91 | 73.45 | 54.54 |
| Health related quality of life | 70.62 | 63.63 | 71.30 | 73.91 |
| Self harm* | 70.39 | 68.18 | 69.37 | 78.95 |
| Lack of cooperation with care | 68.90 | 45.45 | 72.03 | 75.00 |
| Depressive symptoms | 68.12 | 50.00 | 68.70 | 82.61 |
| Family/carer distress | 67.91 | 73.91 | 68.38 | 59.09 |
| Ability to complete ADLs | 64.42 | 65.22 | 68.96 | 41.67 |
| Health/social care resource use | 62.17 | 47.62 | 67.82 | 45.00 |
| Change in continence | 61.87 | 33.34 | 64.10 | 77.27 |
| Happiness/life satisfaction | 61.87 | 54.54 | 60.87 | 73.91 |
| Delirium subtype | 61.29 | 57.14 | 64.04 | 50.00 |
| Mobility | 60.74 | 52.17 | 66.38 | 41.66 |
| Food intake | 58.60 | 38.10 | 62.28 | 59.09 |
| Weight loss | 56.61 | 39.13 | 59.13 | 61.91 |
| Family perceptions of quality of care | 52.15 | 45.46 | 51.28 | 62.50 |
| Staff distress | 50.62 | 39.13 | 50.43 | 63.64 |
| Engagement with meaningful activities | 49.37 | 45.45 | 50.86 | 45.45 |
| Social interactions | 48.12 | 40.91 | 49.14 | 50.00 |
| Constipation | 46.50 | 10.00 | 53.91 | 40.91 |
| Altered skin integrity | 43.04 | 22.72 | 46.08 | 47.62 |

Supplementary Materials Appendix 3: Outcomes of Consensus Meetings and Voting

| OUTCOME | Decision from consensus meeting 1 | Decision from consensus meeting 2 | Consensus reached? | Voting- N (%) in favour of inclusion | Consensus Decision |
| --- | --- | --- | --- | --- | --- |
| Delirium severity | subordinate to “delirium related distress” | yes | No | 12/17 (71%) | INCLUDE |
| Delirium resolution | Yes | Subordinate to ‘delirium occurrence’ | No | 7/17 (41%) | Exclude |
| Patient Distress | Yes, as “delirium related distress” | yes | Yes | n/a | INCLUDE |
| Agitation occurrence | Subordinate to “delirium related distress” | Subordinate to “delirium related distress” | Yes | n/a | Exclude |
| Delirium duration | Subordinate to “delirium resolution” | Subordinate to ‘delirium occurrence’ | Yes | n/a | Exclude |
| Delirium occurrence | yes | yes | Yes | n/a | INCLUDE |
| Falls | no | no | Yes | n/a | Exclude |
| Number of episodes of delirium | Subordinate to “delirium occurrence” | no | Yes | n/a | Exclude |
| Worsening of underlying dementia | no | no | Yes | n/a | Exclude |
| Sleep | no | no | Yes | n/a | Exclude |
| Staff awareness and/ or understanding of delirium | no | no | Yes | n/a | Exclude |
| Mortality | yes | yes | Yes | n/a | INCLUDE |
| Medication appropriateness | no | no | Yes | n/a | Exclude |
| Use of antipsychotic or sedative medication | Subordinate to “delirium related distress” | no | Yes | n/a | Exclude |
| Aggression | Subordinate to “delirium related distress” | Subordinate to “delirium related distress” | Yes | n/a | Exclude |
| Psychotic symptoms | Subordinate to “delirium related distress” | yes | No | 5/17 (29%) | Exclude |
| Cognition including memory | no | yes | No | 9/15 (60%) | INCLUDE |
| Fluid intake | no | no | Yes | n/a | Exclude |
| Infection | no | no | Yes | n/a | Exclude |
| Pain | no | no | Yes | n/a | Exclude |
| Admission to hospital | yes | no | No | 10/17 (59%) | INCLUDE |
| Quality of interpersonal communication | No | No | yes | n/a | Exclude |
| Polypharmacy | No | No | Yes | n/a | Exclude |
| Health related quality of life | No | No | Yes | n/a | Exclude |
| Lack of cooperation with care and treatment | Subordinate to “delirium related distress” | No | Yes | n/a | Exclude |
