# Supplementary material for: Core outcome set for studies evaluating interventions to prevent or treat delirium in long-term care older residents: international key stakeholder informed consensus study
Source: Age Ageing. 2024 Oct 13;53(10):afae227. doi: 10.1093/ageing/afae227 (PMC11471312; doi:10.1093/ageing/afae227)
Supplement: Supplementary_Materials_Appendix_2_afae227 [file supplementary_materials_appendix_2_afae227.docx]

Supplementary Materials Appendix 2: Results from Delphi Round 1

| Outcomes (% rating critical to include) | Overall | Researcher  (N=24) | Clinician  (N=121) | Family  (N=24) |
| --- | --- | --- | --- | --- |
| Patient distress | 91.36 | 100.00 | 90.60 | 86.36 |
| Delirium severity | 88.68 | 86.36 | 89.57 | 86.36 |
| Agitation occurrence | 84.57 | 86.96 | 86.33 | 72.73 |
| Mortality | 84.41 | 81.82 | 83.33 | 94.44 |
| Delirium resolution | 84.28 | 81.82 | 86.96 | 72.73 |
| Delirium duration | 83.65 | 86.36 | 84.35 | 77.27 |
| Public awareness of delirium | 82.91 | 68.18 | 82.61 | 100.00 |
| Staff awareness of delirium | 82.91 | 68.18 | 82.61 | 100.00 |
| Aggression | 82.71 | 65.22 | 80.33 | 81.82 |
| Falls | 82.39 | 72.73 | 84.35 | 81.82 |
| Psychotic symptoms | 81.76 | 72.73 | 82.61 | 86.36 |
| Delirium occurrence | 81.65 | 86.36 | 81.58 | 77.27 |
| Cognition including memory | 81.24 | 81.81 | 84.35 | 60.87 |
| Development/worsening of dementia | 80.62 | 72.73 | 83.35 | 69.57 |
| Pain | 80.62 | 69.57 | 84.35 | 72.73 |
| Number of delirium episodes | 79.87 | 81.82 | 80.00 | 77.27 |
| Fluid intake | 76.73 | 65.22 | 79.13 | 76.19 |
| Use of antipsychotic medication | 75.95 | 81.81 | 77.39 | 61.90 |
| Medication appropriateness | 75.93 | 72.72 | 77.78 | 69.57 |
| Infection | 75.77 | 52.17 | 81.58 | 70.83 |
| Sleep | 74.54 | 72.72 | 77.59 | 60.87 |
| Quality of interpersonal communications | 73.01 | 63.63 | 73.73 | 78.26 |
| Polypharmacy | 71.34 | 61.90 | 79.31 | 35.00 |
| Admission to hospital | 70.89 | 73.91 | 73.45 | 54.54 |
| Health related quality of life | 70.62 | 63.63 | 71.30 | 73.91 |
| Self harm* | 70.39 | 68.18 | 69.37 | 78.95 |
| Lack of cooperation with care | 68.90 | 45.45 | 72.03 | 75.00 |
| Depressive symptoms | 68.12 | 50.00 | 68.70 | 82.61 |
| Family/carer distress | 67.91 | 73.91 | 68.38 | 59.09 |
| Ability to complete ADLs | 64.42 | 65.22 | 68.96 | 41.67 |
| Health/social care resource use | 62.17 | 47.62 | 67.82 | 45.00 |
| Change in continence | 61.87 | 33.34 | 64.10 | 77.27 |
| Happiness/life satisfaction | 61.87 | 54.54 | 60.87 | 73.91 |
| Delirium subtype | 61.29 | 57.14 | 64.04 | 50.00 |
| Mobility | 60.74 | 52.17 | 66.38 | 41.66 |
| Food intake | 58.60 | 38.10 | 62.28 | 59.09 |
| Weight loss | 56.61 | 39.13 | 59.13 | 61.91 |
| Family perceptions of quality of care | 52.15 | 45.46 | 51.28 | 62.50 |
| Staff distress | 50.62 | 39.13 | 50.43 | 63.64 |
| Engagement with meaningful activities | 49.37 | 45.45 | 50.86 | 45.45 |
| Social interactions | 48.12 | 40.91 | 49.14 | 50.00 |
| Constipation | 46.50 | 10.00 | 53.91 | 40.91 |
| Altered skin integrity | 43.04 | 22.72 | 46.08 | 47.62 |
