# Supplementary material for: Core outcome set for studies evaluating interventions to prevent or treat delirium in long-term care older residents: international key stakeholder informed consensus study
Source: Age Ageing. 2024 Oct 13;53(10):afae227. doi: 10.1093/ageing/afae227 (PMC11471312; doi:10.1093/ageing/afae227)
Supplement: Supplementary_Materials_Appendix_3_afae227 [file supplementary_materials_appendix_3_afae227.docx]

Supplementary Materials Appendix 3: Outcomes of Consensus Meetings and Voting

| OUTCOME | Decision from consensus meeting 1 | Decision from consensus meeting 2 | Consensus reached? | Voting- N (%) in favour of inclusion | Consensus Decision |
| --- | --- | --- | --- | --- | --- |
| Delirium severity | subordinate to “delirium related distress” | yes | No | 12/17 (71%) | INCLUDE |
| Delirium resolution | Yes | Subordinate to ‘delirium occurrence’ | No | 7/17 (41%) | Exclude |
| Patient Distress | Yes, as “delirium related distress” | yes | Yes | n/a | INCLUDE |
| Agitation occurrence | Subordinate to “delirium related distress” | Subordinate to “delirium related distress” | Yes | n/a | Exclude |
| Delirium duration | Subordinate to “delirium resolution” | Subordinate to ‘delirium occurrence’ | Yes | n/a | Exclude |
| Delirium occurrence | yes | yes | Yes | n/a | INCLUDE |
| Falls | no | no | Yes | n/a | Exclude |
| Number of episodes of delirium | Subordinate to “delirium occurrence” | no | Yes | n/a | Exclude |
| Worsening of underlying dementia | no | no | Yes | n/a | Exclude |
| Sleep | no | no | Yes | n/a | Exclude |
| Staff awareness and/ or understanding of delirium | no | no | Yes | n/a | Exclude |
| Mortality | yes | yes | Yes | n/a | INCLUDE |
| Medication appropriateness | no | no | Yes | n/a | Exclude |
| Use of antipsychotic or sedative medication | Subordinate to “delirium related distress” | no | Yes | n/a | Exclude |
| Aggression | Subordinate to “delirium related distress” | Subordinate to “delirium related distress” | Yes | n/a | Exclude |
| Psychotic symptoms | Subordinate to “delirium related distress” | yes | No | 5/17 (29%) | Exclude |
| Cognition including memory | no | yes | No | 9/15 (60%) | INCLUDE |
| Fluid intake | no | no | Yes | n/a | Exclude |
| Infection | no | no | Yes | n/a | Exclude |
| Pain | no | no | Yes | n/a | Exclude |
| Admission to hospital | yes | no | No | 10/17 (59%) | INCLUDE |
| Quality of interpersonal communication | No | No | yes | n/a | Exclude |
| Polypharmacy | No | No | Yes | n/a | Exclude |
| Health related quality of life | No | No | Yes | n/a | Exclude |
| Lack of cooperation with care and treatment | Subordinate to “delirium related distress” | No | Yes | n/a | Exclude |
